# DNA damage response alterations in clear cell renal cell carcinoma: clinical, molecular, and prognostic implications

**DOI:** 10.1186/s40001-024-01678-x

**Published:** 2024-02-07

**Authors:** Xiao Jing, Xiangcheng Qin, Hao Liu, Huanhuan Liu, Huina Wang, Jiayue Qin, Yanui Zhang, Shanbo Cao, Xiaodong Fan

**Affiliations:** 1 Department of Urology, The Second Affiliated Hospital of Zhejiang University School of Medicine, Hangzhou, China; 2 Department of Urology, Ningbo Urology and Nephrology Hospital, Ningbo, China; 3 Acornmed Biotechnology Co., Ltd., Beijing, China

**Keywords:** Clear cell renal cell carcinoma (ccRCC), DNA damage repair (DDR), Prognosis, Tumor microenvironment, Tumor mutational burden (TMB)

## Abstract

**Background:**

DNA damage repair (DDR) pathways modulate cancer risk, progression, and therapeutic responses. Nonetheless, the characteristics and significance of DDR alterations in clear cell renal cell carcinoma (ccRCC) remain undefined. This study aimed to explore the predictive role, molecular mechanism, and tumor immune profile of DDR genes in ccRCC.

**Methods:**

We prospectively sequenced 757 tumors and matched blood DNA samples from Chinese patients with ccRCC using next-generation sequencing (NGS) and analyzed data from 537 patients from The Cancer Genome Atlas (TCGA). A comprehensive analysis was performed.

**Results:**

Fifty-two percent of Chinese patients with ccRCC harbored DDR gene mutations and 57% of TCGA patients. The immunotherapy treatment prognosis of patients with DDR gene mutations was superior to that of patients without DDR gene mutations (*p* = 0.047). DDR gene mutations were associated with more gene mutations and a higher tumor mutation load (TMB, *p* < 0.001). Moreover, patients with DDR gene mutations have a distinct mutational signature compared with those with wild-type DDR. Furthermore, the DDR-mut group had elevated neoantigen load (including single-nucleotide variants (SNV) and indel neoantigen load, *p* = 0.037 and *p* = 0.002, respectively), TCR Shannon (*p* = 0.025), and neutrophils (*p* = 0.010). DDR gene mutations exhibited a distinct immune profile with significantly higher expression levels of TNFSF9, CD70, ICAM1, and indoleamine-2,3-dioxygenase (IDO) and lower expression levels of VTCN1 and IL12A.

**Conclusions:**

Our data suggest that the detection of somatic mutations in DDR genes can predict the efficacy of immunotherapy in patients with ccRCC. Furthermore, we revealed the unique molecular and immune mechanisms underlying ccRCC with DDR gene mutations.

**Supplementary Information:**

The online version contains supplementary material available at 10.1186/s40001-024-01678-x.

## Background

Kidney cancer is one of the most common urological cancers worldwide, with 431,288 newly diagnosed cases and 179,368 cancer-related deaths in 2020 [1]. Renal cell carcinoma (RCC) is the most common solid lesion within the kidney and accounts for approximately 90% of all kidney cancers and 2–3% of all adult malignancies [2]. Clear cell RCC (ccRCC) is the most common RCC histologic subtype [3]. Most RCC are localized and managed with surgery, while metastasis occurs in about 20–40% of localized primary RCC following surgical resection [4–6]. In addition, because of its innate high invasiveness and strong resistance to traditional therapies such as radiotherapy and chemotherapy, there are no effective postoperative adjuvant therapies [7]. Furthermore, there are several major new tools available for RCC management, but large patient cohorts are needed to validate previously obtained results and move into clinical practice [8, 9]. Therefore, it is important to identify new therapeutic targets to improve the prognosis of ccRCC.

For more than 20 years, immunotherapy has been considered a treatment option for metastatic RCC because of the immunogenicity of the tumor and the demonstration of spontaneous regressions [10]. Although immunotherapy has significantly changed the therapeutic landscape in mRCC, the overall clinical effects, including low immune-related adverse events and low response rates, are still unsatisfactory [11]. Therefore, the identification of novel actionable targets and predictors of treatment response is critical for the better management of this disease.

The DNA damage response (DDR) pathway is an intracellular monitoring mechanism that is activated and contributes to maintaining the integrity and stability of the cell genome when DNA damage occurs [12]. Furthermore, DDR deficiency is associated with sensitivity to immunotherapies, which tend to accumulate tumor-specific neoantigens, resulting in a strong anti-tumor immune response [13, 14]. However, the molecular status of the DDR pathway in ccRCC is unclear, and it is undefined how DDR genes affect the cancer treatment effect of immunotherapy. Because of the above information, we analyzed how DDR gene mutations affect the efficacy of immunogenicity therapy in ccRCC patients from the aspects of the tumor genome, tumor immunogenicity, and the tumor immune microenvironment.

## Methods

### Patient enrollment and study design

A total of 757 Chinese patients with ccRCC were recruited from the Second Affiliated Hospital of Zhejiang University School of Medicine and Ningbo Urology and Nephrology Hospital between June 2018 and September 2021. Informed consent was obtained from all the participants. This study was approved by the Ethics Committee of the Second Affiliated Hospital of Zhejiang University School of Medicine and Ningbo Urology and Nephrology Hospital. A total of 537 patients with ccRCC in The Cancer Genome Atlas (TCGA) database were obtained from the cBioPortal data portal (http://www.cbioportal.org/). 35 patients in the immune checkpoint inhibitor-treated cohort were included (MSK-IMPACK cohort).

### DDR pathway

A total of 123 DDR-related genes and 10 DDR functional pathways were covered by our Next Generation Sequencing (NGS) panels, which were referenced in previous reports, including direct repair (DR), mismatch repair (MMR), base excision repair (BER), nucleotide excision repair (NER), Fanconi anemia (FA), translesion synthesis (TLS), non-homologous end joining (NHEJ), homologous recombination (HR), and cell cycle checkpoint (CCC) [15, 16].

### Library preparation and next-generation sequencing

Tissue DNA and blood controls were extracted using the QIAamp Genomic DNA kit (Qiagen, Hilden, Germany) according to our previous report [17]. Sequencing libraries were used according to Illumina standard library construction instructions (Illumina, Inc.). The 808 cancer-related gene panel was also enriched. The sequencing depth was > 10,000×. Sequence reads were aligned to the human reference genome (GRCh37) using the Burrows–Wheeler alignment (BWA) tool [18]. Local realignment and base quality score recalibration were performed using GATK software [19]. Single-nucleotide variants (SNVs) and small insertions or deletions (INDEL) were identified using MuTect2 [20]. Copy number alteration (CNA) calling was analyzed using CONTRA software [21].

### Calculation of the tumor mutational burden (TMB)

TMB was defined as the number of somatic, coding, and INDEL mutations and base substitutions per megabase (Mb) of the genome examined [17]. TMB was calculated as the total number of mutations counted/2 Mb.

### Mutational signature analysis

Mutational signature analysis was performed to resolve SNVs for each sample into a set of characteristic patterns (signatures). SNVs were classified into 96 base-substitution types for each sample by NMF using the R package MutationalPatterns [22, 23]. The signatures discovered were compared with 30 COSMIC signatures. When the similarity values of different signatures were less than 0.80, this signature was defined as a new signature [24].

### Statistical analyses

Statistical analyses were performed using the R package. Fisher’s exact test was performed for comparisons between two categorical variables, and false discovery rate (FDR) correction was performed. The Mann–Whitney *U* test was used to compare two continuous variables. Survival analysis was performed based on Kaplan–Meier survival plots. Probability values were derived from two-sided tests and differences were considered significant at *p* < 0.05.

## Results

### Patient demographic and clinical data

A total of 757 Chinese ccRCC patients were enrolled, including 395 patients with DDR mutation (DDR-mut) and 362 patients with wild-type DDR (DDR-wt). Patient characteristics are summarized in Additional file 5: Table S1. In total, 75.8% of the patients were male. The median age of the included patients was 67 years (range: 25–87). In total, 81.8% of the samples were tissue. Targeted therapy and immunotherapy were administered to 9.3% and 2.0% of patients, respectively. In addition, DDR gene mutations were associated with age (*p* = 0.005). Interestingly, patients with DDR gene mutations received targeted therapy at a lower rate than those without DDR gene mutations (*p* = 0.07). 537 TCGA ccRCC were included in this study. The male-to-female ratio was 1.8. Half of the patients had stage I (50.1%). Most patients were in G2 (42.8%) or G3 (38.6%). Conversely, DDR gene mutations were not associated with age or other clinical information in the TCGA cohort (*p* > 0.05).

### Landscape of DDR gene mutation in the Chinese and TCGA ccRCC

**Fig. 1:**
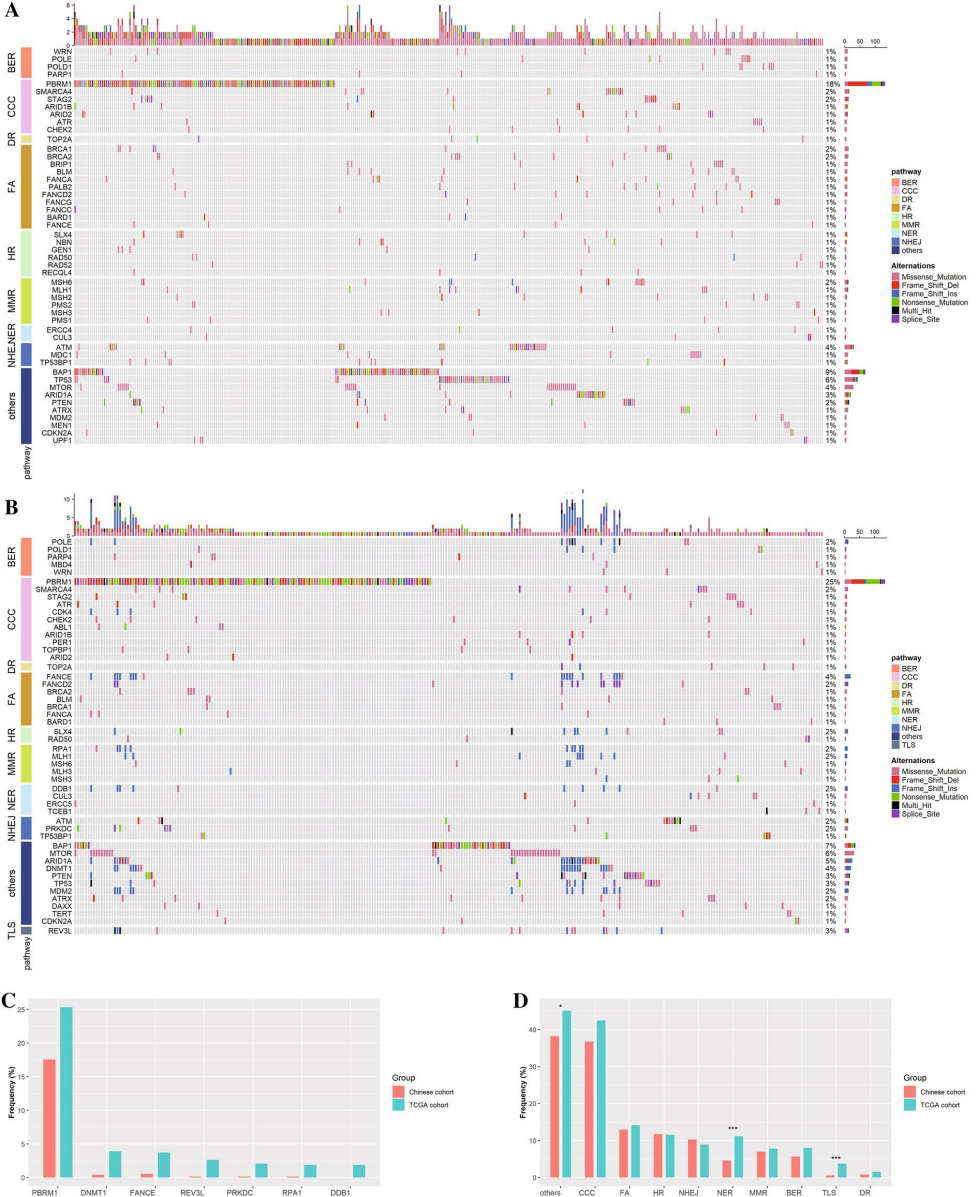
Distribution of DNA damage repair (DDR) gene mutations in patients with ccRCC. Landscape of DDR genes in the Chinese cohort (**A**) and TCGA cohort (**B**). The columns and rows represent patients and genes. Frequency of DDR mutated genes (right), and mutation type for each sample (right). **C** Significant differences in variant allele frequencies for DDR genes between the Chinese cohort and TCGA cohort. **D** Distribution of patients with mutations in the 10 DDR functional pathways in both the Chinese cohort and TCGA cohort

We explored the DDR mutation profile in ccRCC. A total of 758 DDR gene mutations were noted in 52.3% (395/757) of Chinese patients. The most frequently mutated genes were PBRM1 (17.6%), BAP1 (9.0%), TP53 (5.7%), ATM (4.0%), and MTOR (3.8%) (Fig. 1A). In the TCGA cohort, 756 DDR gene mutations were mapped in 56.6% (304/537) of the patients. The most frequently mutated genes were PBRM1 (25.3%), BAP1 (6.7%), MTOR (5.8%), ARID1A (4.7%), and DNMT1 (3.9%) (Fig. 1B). There were no significant differences in the frequency of DDR-mutated genes between the two cohorts. Compared to TCGA cohort, there was a lower frequency of PBRM1 (FDR = 0.016), DNMT1 (FDR < 0.001), FANCE (FDR = 0.001), REV3L (FDR = 0.010), PRKDC (FDR = 0.010), RPA1 (FDR = 0.016), and DDB1 (FDR = 0.004) in the Chinese cohort (Fig. 1C). Among the 10 functional pathways of DDR genes, the most commonly mutated pathways were the others, CCC, and FA pathways in both TCGA and Chinese cohorts (Fig. 1D). Furthermore, the Chinese cohort had a lower frequency of others (FDR = 0.046), NFR (FDR < 0.001), and TLS pathways (FDR < 0.001) than those in the TCGA cohort.

### DDR gene reveals immunotherapy prognosis

**Fig. 2:**
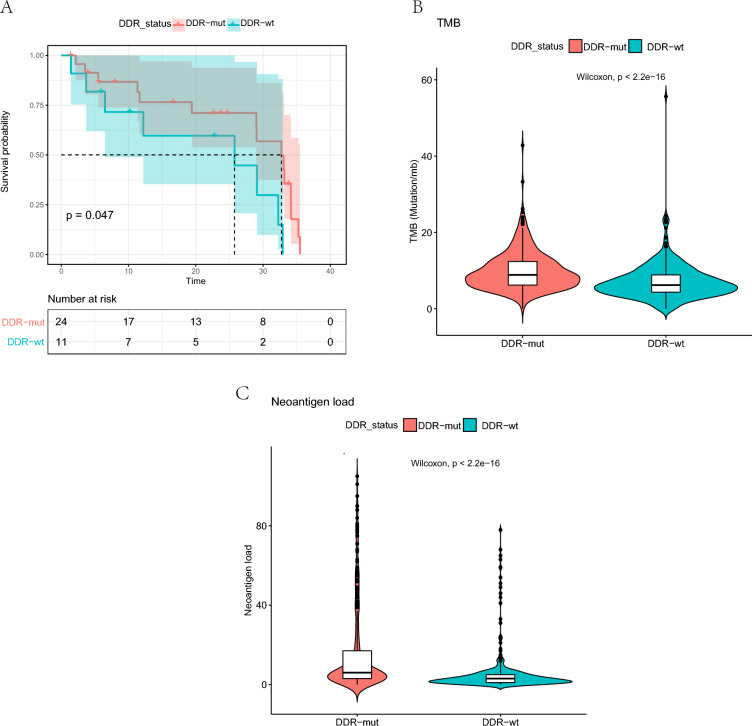
Feasibility of DDR gene mutations in predicting the response to immunotherapy. **A** Overall survival of patients stratified by DDR-mut/wt status in the immunotherapy cohort. **B** The relationship between DDR status and tumor mutational burden (TMB). **C** The relationship between DDR status and neoantigen load

To explore the predictive value of DDR gene mutations in predicting ccRCC prognosis, we investigated the relationship between DDR gene mutations and clinical outcomes using TCGA data. Unfortunately, DDR gene mutations did not predict OS (*p* = 0.770; Additional file 1: Fig. S1A) or PFS (*p* = 0.170; Additional file 1: Fig. S1B). Notably, patients receiving immunotherapy with DDR gene mutations had longer OS than those receiving wild-type DDR (*p* = 0.047, Fig. 2A). However, DDR gene mutations were not associated with PFS in patients who received immunotherapy. Subsequently, we further explored the relationship between DDR and immunotherapy in Chinese patients with renal cancer by analyzing the correlation between DDR and immunotherapy markers. We found that DDR gene mutations were associated with higher tumor mutational burden (TMB) (*p* < 0.001, Fig. 2B) and neoantigen load (*p* < 0.001, Fig. 2C) in Chinese ccRCC patients, which may indicate that DDR is associated with immunotherapy response in the Chinese population.

### Differences in genomic patterns of ccRCC patients with different DDR status

**Fig. 3:**
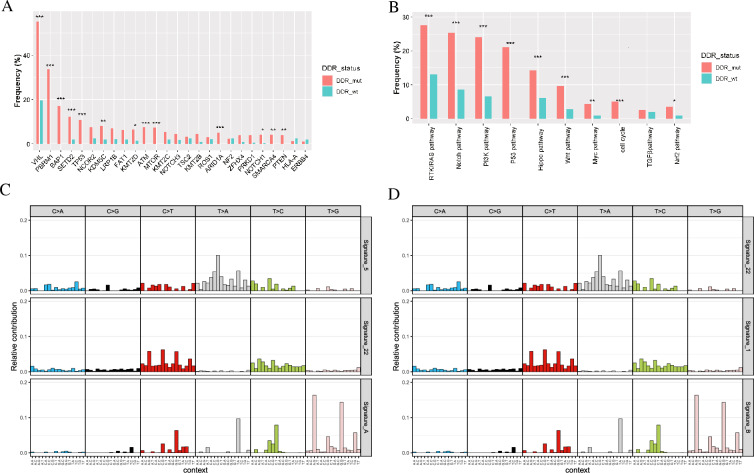
Differences in molecular mechanism between the DDR-mut group and DDR-wt group. **A** Differences in gene profiles between two groups. **B** Differences in oncopathways between two groups. **C** Mutational signatures in DDR-mut cohorts. **D** Mutational signatures in DDR-wt cohorts

To better understand how DDR affects genomic patterns in ccRCC, we analyzed somatic mutations and CNA between patients with DDR-mut and DDR-wt. The most frequently mutated genes in the Chinese DDR-mut group were VHL (55.2%), PBRM1 (33.7%), and BAP1 (17.2%) (Additional file 2: Fig. S2A). The most frequently mutated genes in the DDR-wt group were VHL (19.6%), NCOR2 (2.5%), and HLA-A (2.5%) (Additional file 2: Fig. S2B). 22 genes were significantly different between the DDR-mut and DDR-wt groups (FDR < 0.05) (Additional file 6: Table S2). Among the top 20 genes, 13 genes in the DDR-mut group had significantly higher mutation frequencies than those in the DDR-wt group, including VHL, NCOR2, BRD4, and FAT1 (Fig. 3A). Similarly, in the TCGA cohort, the most frequently mutated genes were VHL (51.6%) and PBRM1 (45.1%) in patients with DDR gene mutations (Additional file 2: Fig. S2C), and the most frequently mutated genes were VHL (29.2%) and MUC (9.4%) in patients without DDR gene mutations (Additional file 2: Fig. S2D). 69 genes were significantly different between the DDR-mut and DDR-wt groups in the TCGA cohort (FDR < 0.05) (Additional file 7: Table S3). In addition, the most common signaling pathways enriched in both groups were the RTK/RAS, Notch, and PI3K pathways in Chinese cohorts (Fig. 3B). Furthermore, patients with DDR gene mutations were more likely to harbor mutations in genes involved in oncopathways than those with wild-type DDR (Fig. 2B).

### Relationship between mutational signature and HRR status

To better comprehend the contribution of these mutations to the DDR status, an in-depth exploration based on mutational signatures was conducted. There were three known signatures [signature 1 for aging, signature 5 for ERCC2 mutations, and signature 22 exposure to aristolochic acid (AA)] and two de novo mutational signatures, named signatures A and B, obtained by extracting mutation catalogs from Chinese ccRCC patients. Only signature 22 was found in both groups. Signatures 5 and A were observed in the DDR-mut group (Fig. 3C). Furthermore, signature 5 was the main signature type in patients with DDR gene mutations, accounting for 55.3%, followed by signatures A (23.1%), and 22 (21.5%) (Additional file 3: Fig. S3A). Similarly, signature 1 for the spontaneous deamination of 5-methylcytosine and signature B were observed only in the DDR-wt group (Fig. 3D). Signature 1 accounted for the highest proportion in the DDR-wt group (53.6%), whereas signature 22 had a similar proportion in the DDR-wt group (22.3%, Additional file 3: Fig. S3A).

### Co-occurrence and mutual exclusion among genetic events

**Fig. 4:**
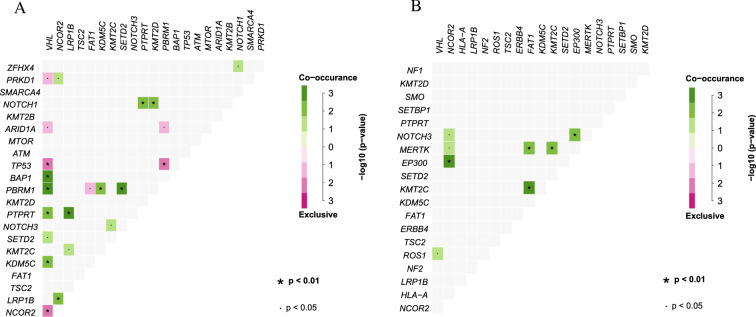
Correlation between observed and expected co-occurrence of mutations in the DDR-mut group (**A**) and DDR-wt group (**B**)

To identify correlations among the different genes in the two groups, co-occurrence and mutual exclusion analyses of the top 20 genes in each group were performed. Genetic interactions were markedly different between the two groups (Fig. 4A, B). Mutations in NOTCH3 and KMT2C were significantly associated (co-occurring) in the DDR-mut group (Fig. 4A). However, no co-occurrence was observed in the HRR-wt group (Fig. 4A). Similarly, VHL co-occurred with BAP1, and PTPRT, and was mutually exclusive with TP53 and ARID1A mutations in the DDR-mut group (Fig. 4A). However, this correlation was not observed in the DDR-wt group (Fig. 4B).

### DDR gene mutations increased genomic instability

Previous studies have reported that DDR gene mutations increase genomic instability; Therefore, we further explored whether DDR has the same contribution in ccRCC [25]. In this study, Chinese patients with DDR gene mutations had higher wGII (*p* < 0.001) and ITH scores (*p* < 0.001) than patients without DDR gene mutations (Additional file 3: Fig. S3B). Similarly, in the TCGA cohort, DDR gene mutations also increased genomic instability, including high ITH (*p* = 0.008), TMB (*p* < 0.001), silent mutation rate (*p* = 0.006), and nonresilient mutation rate (*p* = 0.0001) (Additional file 3: Fig. S3C). In addition, although other genomic instability indicators (Fraction Altered: *p* = 0.06; Aneuploidy Score: *p* = 0.06) did not show significant differences, an increasing trend was observed in the DDR-mut group (Additional file 3: Fig. S3B).

### DDR gene mutations enhance immunogenicity

**Fig. 5:**
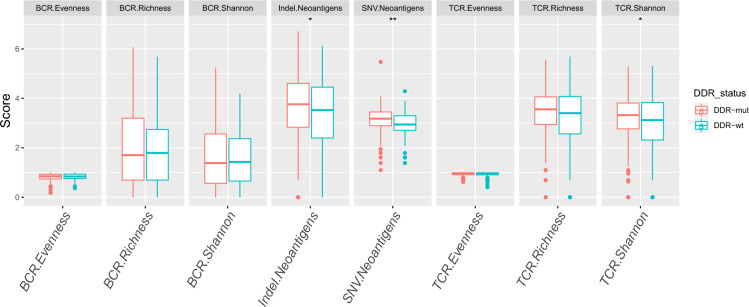
Relationship between DDR status and immunogenicity. Evaluation of neoantigen load, B-cell receptor (BCR) richness, and T-cell receptor (TCR) richness using −log(neoantigen load), −log(BCR richness), and −log(TCR richness)

A growing body of evidence supports the concept that DDR gene mutations can increase the anti-tumor immune response by promoting antigenicity through increased mutability and genomic instability [25]. We further explored the relationship between DDR gene mutations and immunogenicity. It is difficult to measure the actual immunogenicity of tumors; however, some basic parameters, including tumor antigenicity and the efficiency of antigen processing and presentation, can be used to assess immunogenicity. We discovered that patients with DDR gene mutations had a higher neoantigen load (including SNV and indel neoantigen loads) than those without DDR gene mutations (*p* = 0.037 and *p* = 0.002, respectively; Fig. 5). In addition, the DDR-mut group had a higher TCR Shannon index than that of the DDR-wt group (*p* = 0.025). However, DDR gene status was not associated with antigen-specific B-cell receptor (BCR) repertoires (Fig. 5).

### DDR gene mutation status associated with immune cells and immune regulatory gene expression profiles

**Fig. 6:**
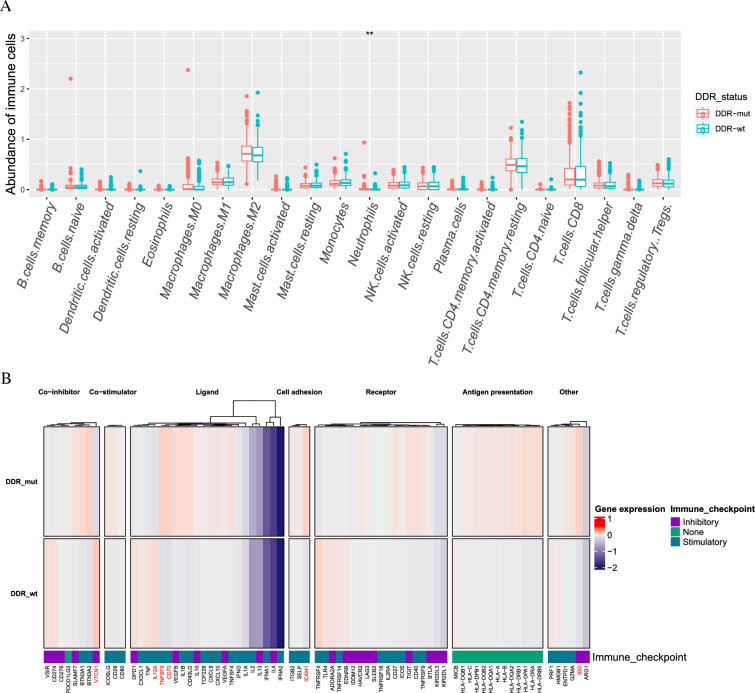
Differences in immune cells and immune-related genes between the DDR-mut and DDR-wt groups. **A** The relationship between immune cells and DDR mutation status. **B** The relationship between immune-related genes and DDR mutation status

To further explore the role of DRR mutations in immune activity, we analyzed the relationship between DDR status and immune cells. DDR gene mutations were associated with elevated neutrophil levels (*p* = 0.010; Fig. 6A). However, DDR was not associated with other immune cells (*p* > 0.05). We then investigated whether the DDR mutation status affected immune-regulatory gene expression. As shown in Figs. 6B and Additional file 4: Fig. S4, these two groups of patients presented a significantly different expression landscape for immunomodulators. In particular, ligand and cell adhesion-related gene expression, including TNFSF9 (*p* = 0.019), CD70 (*p* = 0.003), and ICAM1 (*p* = 0.011), was higher in the DDR-mut group than in the DDR-wt group. Moreover, patients with DDR gene mutations harbored fewer VTCN1 genes for co-inhibitor roles than patients without DDR gene mutations (*p* = 0.004). In addition, DDR-mut was associated with elevated indoleamine-2,3-dioxygenase (IDO) (*p* = 0.038) and downregulation of IL12A (*p* = 0.043).

## Discussion

The discovery of immunotherapy has changed the treatment of ccRCC patients. However, only a few patients completely or partially respond to immunotherapy [26]. Therefore, Screening populations that are more likely to benefit from immunotherapy is urgently required. DDR gene mutations are useful for assessing the response to immune checkpoint blockade therapy and platinum-based chemotherapy in several cancers [13, 27]. Nonetheless, the molecular mechanism and tumor immune profile of DDR genes and their relevance to the clinical therapeutic response in ccRCC remain unclear. To the best of our knowledge, this is the first study to elucidate the comprehensive and profound links between DDR gene mutations and gene mutation characteristics, genomic instability, tumor immunogenicity, abundance of immune cells, and immune-related genes in ccRCC. We also discovered that DDR pathway scores can predict the response to immunotherapy in ccRCC.

DNA damage can induce metabolic rewiring, such as activated DDR triggers an increase in nucleotide synthesis and anabolic glucose metabolism, while also reducing glutamine anaplerosis [28]. Previous studies have confirmed that RCC is essentially a metabolic disease characterized by a reprogramming of energetic metabolism [29–32]. Especially, mitochondrial bioenergetics, OxPhox, and lipid metabolism are impaired, and the metabolic flux through glycolysis is partitioned [33–38]. Thus, we speculated that the mutations in genes involved in the DDR and DNA repair also lead to metabolic rewiring for tumor cells of ccRCC, which promotes the occurrence and progression of ccRCC. In this study, we found that more than 50% of patients with ccRCC carried the DDR mutation including 52.5% of Chinese patients and 56.6% of TCGA patients. This result may imply that at least 50% of the metabolic recombination occurring in ccRCC is related to DDR gene mutations.

Previous studies have confirmed that DDR gene mutations are associated with favorable immunotherapy survival in several cancers [13, 27, 39]. Similarly, in this study, we demonstrated that ccRCC patients with DDR gene mutations may have prolonged survival intervals in immunotherapy settings. However, there is conflicting evidence regarding the clinical predictive efficacy of immunotherapy biomarkers in different cancer subtypes [40]. Zhang et al. reported that the immunotherapy treatment prognosis of bladder cancer (BLCA) and colorectal cancer (CRC) patients with DDR gene mutations was superior to that of patients without DDR gene mutations, while this correlation was not found in the other five cancers [i.e., brain glioma (BG), head and neck cancer (HNSC), non-small cell lung cancer (NSCLC), renal cell carcinoma (RCC), and melanoma (SKCM)] [40]. These inconsistent results may be attributed to differences in DDR gene selection. Several reports have demonstrated that high TMB is associated with the preferable efficacy of immunotherapy [41]. In this study, we found that Chinese patients with DDR gene mutations harbored a higher TMB than Chinese patients without DDR gene mutations, which may imply that DDR gene mutations can also predict the efficacy of immunotherapy in Chinese ccRCC.

Analysis of the genetic landscape of DDR genes in cancer will have important implications for tumor diagnosis, individualized therapy, and targeted drug use [42, 43]. There were significant differences in the mutational landscapes of the DDR-mut and DDR-wt groups, suggesting different mechanisms of carcinogenesis. Furthermore, DDR gene mutations were accompanied by aggregating somatic mutations in our study, which may explain why DDR gene mutations lead to elevated TMB. More importantly, these results imply that TMB may be the outcome, not the driving factor, of oncogenic alterations in ccRCC. Mutational signature analyses provide a method to identify potential mutation sources and mechanisms [23, 44]. In this study, signatures 5 and A were observed only in DDR-mut cohorts. These results imply that these signatures can serve as novel readouts of DDR and contribute to defining patients with ccRCC with DDR gene mutations. In addition, differences in gene interactions demonstrate a unique molecular mechanism in patients with DDR gene mutations. DDR determines tumor antigenic and mutational processes that shape the tumor antigenic landscape [25]. In this study, we discovered that DDR gene mutations were associated with enhanced neoantigen load and TCR Shannon, which may suggest that DDR defects provide a readily available reservoir of neoepitopes, drive elevated tumor neoantigen load, and increase immunogenicity [25]. In addition, we found that DDR gene mutations are significantly associated with genomic instability, which confirms previous studies showing that DDR defects promote antigenicity by increasing mutability and genomic instability [25, 45]. TCR Shannon limits immune escape by increasing the possibility of tumor-specific T cells [46]. In this study, the DDR gene mutations harbored high TCR Shannon values, which may imply that DDR gene mutations increase immunogenicity via multiple pathways, thereby promoting anti-tumor responses.

The characteristics of the tumor microenvironment significantly influence disease biology and may influence response to systemic therapies [47–50]. Furthermore, ccRCC is one of the most immune-infiltrated tumors [51, 52]. Thus, a comprehensive analysis of the association between DDR gene mutations and immune cells is a key step in understanding the mechanisms by which DDR influences prognosis in ccRCC. In the present study, we found that DDR gene mutations were significantly associated with elevated neutrophil count. Anti-tumor neutrophils can directly kill tumor cells, promote T-cell activation, and recruit proinflammatory macrophages [53]. However, the number of T cells and macrophages did not increase in the DDR-mut group. These results suggest that DDR gene mutations play an anti-tumor role by activating the ability of neutrophils to kill tumors in ccRCC. Furthermore, DDR-mut groups showed upregulated expression of TNFSF9, CD70, ICAM-1, and IDO and downregulated IL12A and VTCN1. TNFSF9 and CD70 belong to the tumor necrosis receptor superfamily, which can regulate neutrophils [54, 55]. ICAM-1 mediates neutrophil recruitment, phagocytosis, adhesion, and transepithelial migration [56, 57]. IDO, the main inducible and rate-limiting tryptophan-degrading enzyme, has recently been shown to increase neutrophil infiltration into tumors [58]. Upregulation of the expression of these genes may recruit more neutrophils to provoke an anti-tumor immune response and enhance the response of ccRCC with DDR gene mutations to immunotherapy. In addition, ccRCC is one of the most immune-infiltrated tumors and is essentially a metabolic disease characterized by reprogramming of energetic metabolism [29, 51, 52]. DNA damage can induce metabolic rewiring [28]. The activation of specific metabolic pathways plays an important role in regulating angiogenesis and inflammatory signatures [38, 59]. A reasonable explanation is that DDR gene mutation affects immune function by inducing metabolic recombination and regulating neutrophil activation.

Although this study revealed important discoveries, there also have limitations. First, this study is limited owing to the lack of data on patients receiving immunotherapy from the Chinese groups, which made it impossible to verify the findings obtained with the TCGA groups in the Chinese groups. Second, this study is specifically on the Chinese population, which could limit generalizability of the data. Third, we defined DDR gene mutations as nonsilent mutations in the coding region, which might miss other silent mutations that might affect the function of the protein. However, we might not identify specific protein functions in this study. Fourth, the study was retrospective and might have been prone to unknown bias. Fifth, germline mutations were not included in this study, which might have influenced our results. Finally, we did not distinguish between biallelic and monoallelic inactivation of DDR genes, which might exhibit different degrees of immunogenicity in ccRCC. We will continue to explore the mechanism and application of DDR in ccRCC patients to provide data for the accurate treatment of these patients.

## Conclusions

Our findings might provide insights into biomarker development for further stratified treatment and management of patients with ccRCC. We also demonstrated that DDR gene mutations are prevalent in ccRCC and exhibit distinct immune profiles. In addition, the molecular mechanism of DDR might provide new opportunities to predict tumor responses to multiple treatments.

### Supplementary Information

**Additional file 1: Figure S1.** The relationship between DDR mutation and clinical outcome in the TCGA cohort. (A) Overall survival of patients stratified by DDR-mut/wt status in all patients. (B) Progression-free survival of patients stratified by DDR-mut/wt status in all patients. (C) Progression-free survival of patients stratified by DDR-mut/wt status in the immunotherapy cohort.

**Additional file 2: Figure S2.** Mutational landscape between DDR-mut (A) and DDR-wt group (B).

**Additional file 3: Figure S3.** Molecular characteristics of DDR gene mutations. (A) The proportion of signatures in various DDR status. (B) The relationship between genomic instability and DDR status in the Chinses cohort. (C) The relationship between genomic instability and DDR status in the Chinses cohort. Evaluation of TMB, nonsilent mutation, and number of segments using −log(TMB), −log(nonsilent mutation), and −log(number of segments).

**Additional file 4: Figure S4.** Immune-related genes were significantly differentially expressed in DDR-mut and DDR-wt.

**Additional file 5: Table S1.** Characteristics of Chinese kidney cancer patients.

**Additional file 6: Table S2.** The significantly different gene between the DDR-mut and DDR-wt groups in Chinese cohort.

**Additional file 7: Table S3.** The significantly different gene between the DDR-mut and DDR-wt groups in TCGA cohort.

## Data Availability

The datasets used and/or analyzed during the current study are available from the corresponding author on reasonable request.

## Abbreviations

DDR: DNA damage repair
ccRCC: Clear cell renal cell carcinoma
NGS: Next-generation sequencing
TCGA: The Cancer Genome Atlas
TMB: Tumor mutation load
DR: Direct repair
MMR: Mismatch repair
BER: Base excision repair
NER: Nucleotide excision repair
FA: Fanconi anemia
TLS: Translesion synthesis
NHEJ: Nonhomologous end joining
HR: Homologous recombination
CCC: Cell-cycle checkpoint
SNVs: Single-nucleotide variants
INDELs: Insertions or deletions
CAN: Copy number alteration
FDR: False discovery rate
DDR-mut: DDR mutation
Ddr-wt: Wild-type DDR
