# Supplementary figures and images for: DNA damage response alterations in clear cell renal cell carcinoma: clinical, molecular, and prognostic implications
Source: Eur J Med Res. 2024 Feb 7;29:107. doi: 10.1186/s40001-024-01678-x (PMC10848511; doi:10.1186/s40001-024-01678-x)

A

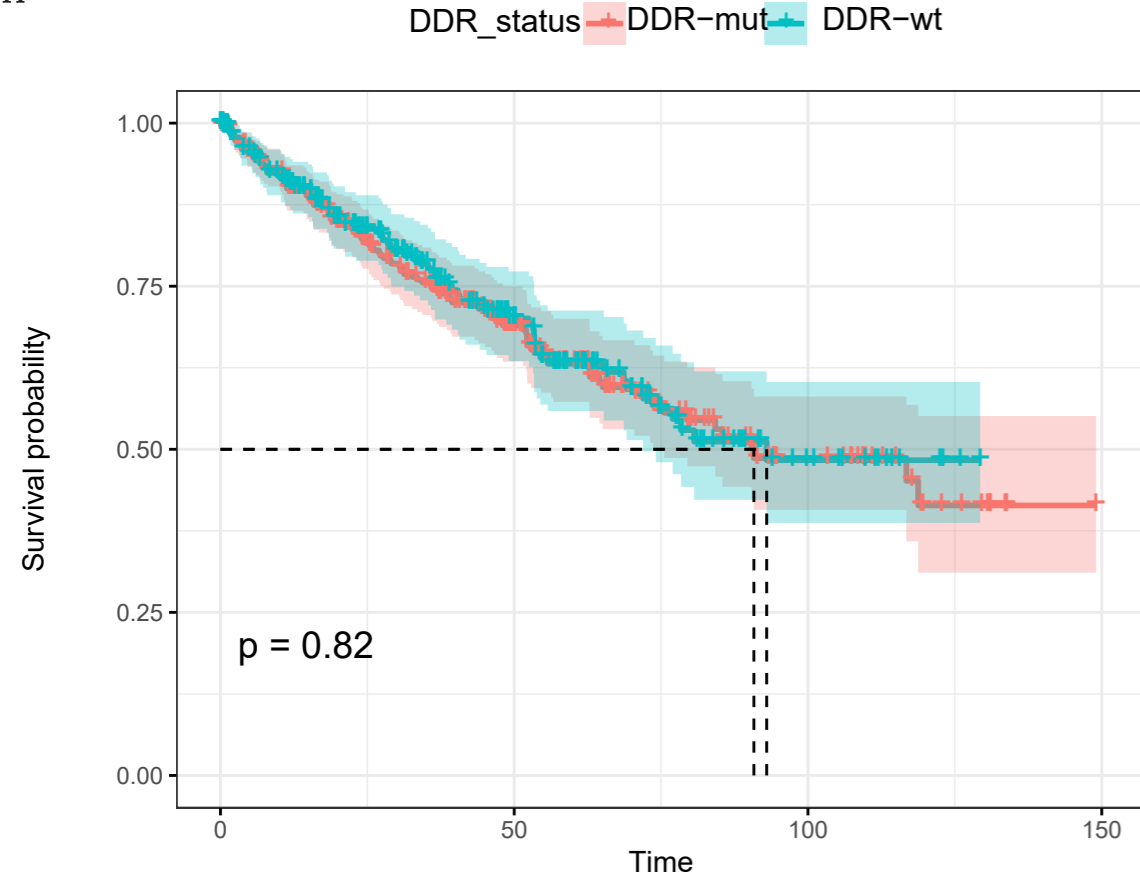

|                |     |     |    |   |
|----------------|-----|-----|----|---|
| Number at risk |     |     |    |   |
| DDR-mut        | 301 | 118 | 25 | 0 |
| DDR-wt         | 236 | 83  | 14 | 0 |

B

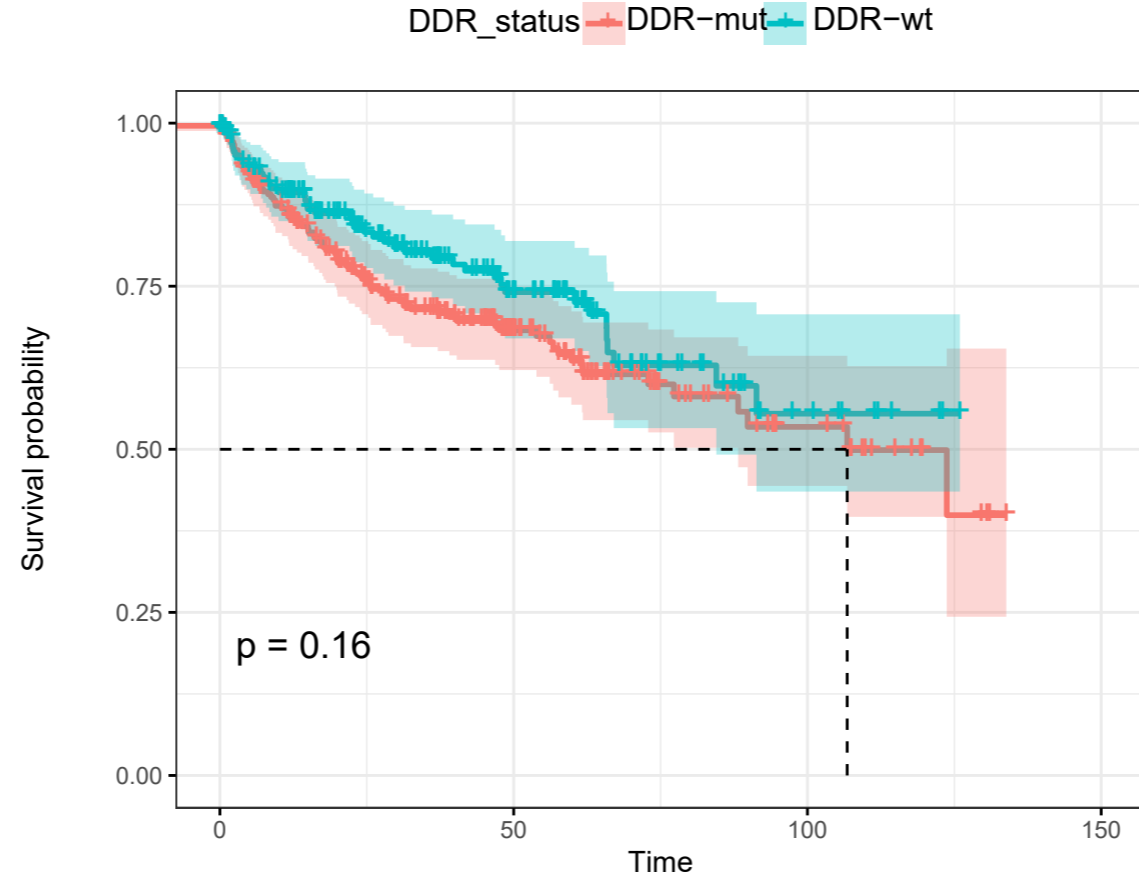

|                |     |    |    |   |
|----------------|-----|----|----|---|
| Number at risk |     |    |    |   |
| DDR-mut        | 245 | 83 | 17 | 0 |
| DDR-wt         | 191 | 62 | 10 | 0 |

C

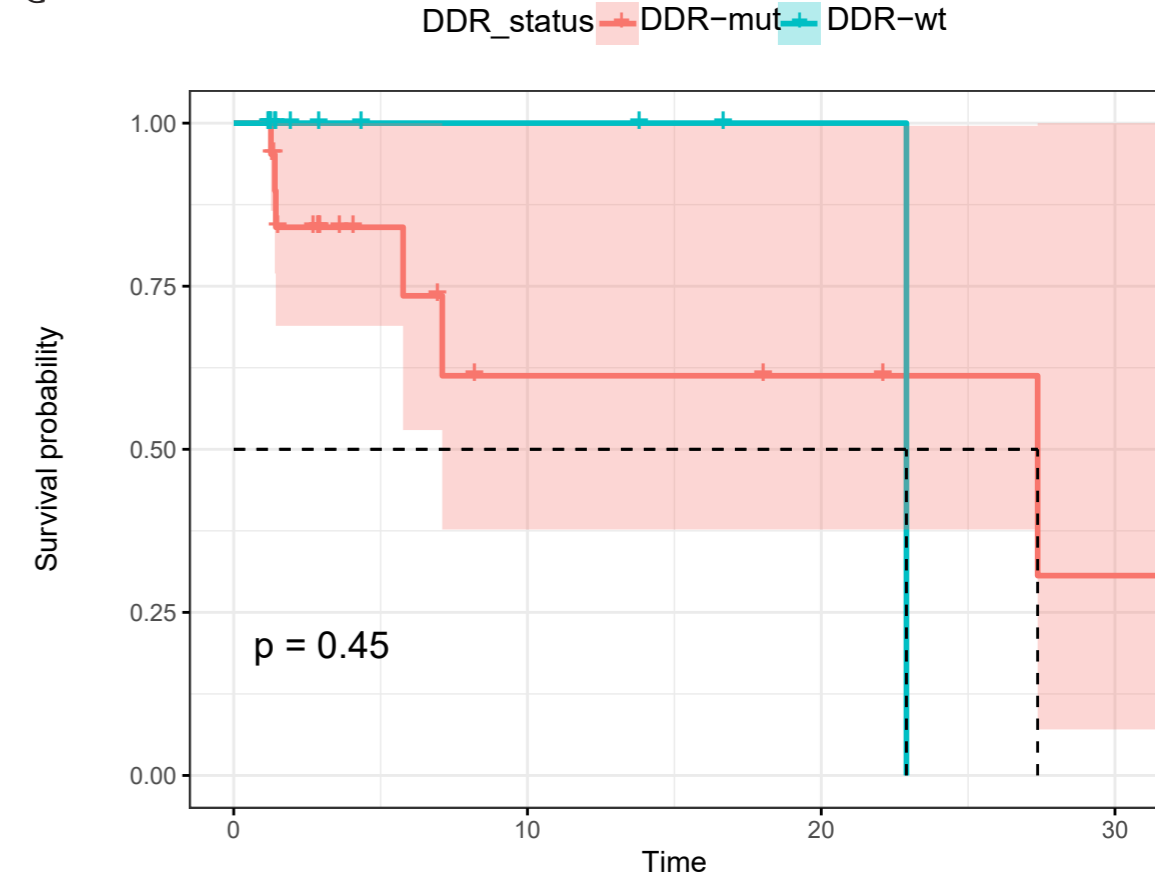

|                |    |   |   |   |
|----------------|----|---|---|---|
| Number at risk |    |   |   |   |
| DDR-mut        | 24 | 4 | 3 | 1 |
| DDR-wt         | 11 | 3 | 1 | 0 |

Supplement: Supplementary file 1 — Additional file 1: Figure S1. The relationship between DDR mutation and clinical outcome in the TCGA cohort. (A) Overall survival of patients stratified by DDR-mut/wt status in all patients. (B) Progression-free survival of patients stratified by DDR-mut/wt status in all patients. (C) Progression-free survival of patients stratified by DDR-mut/wt status in the immunotherapy cohort. [file 40001_2024_1678_MOESM1_ESM.pdf]

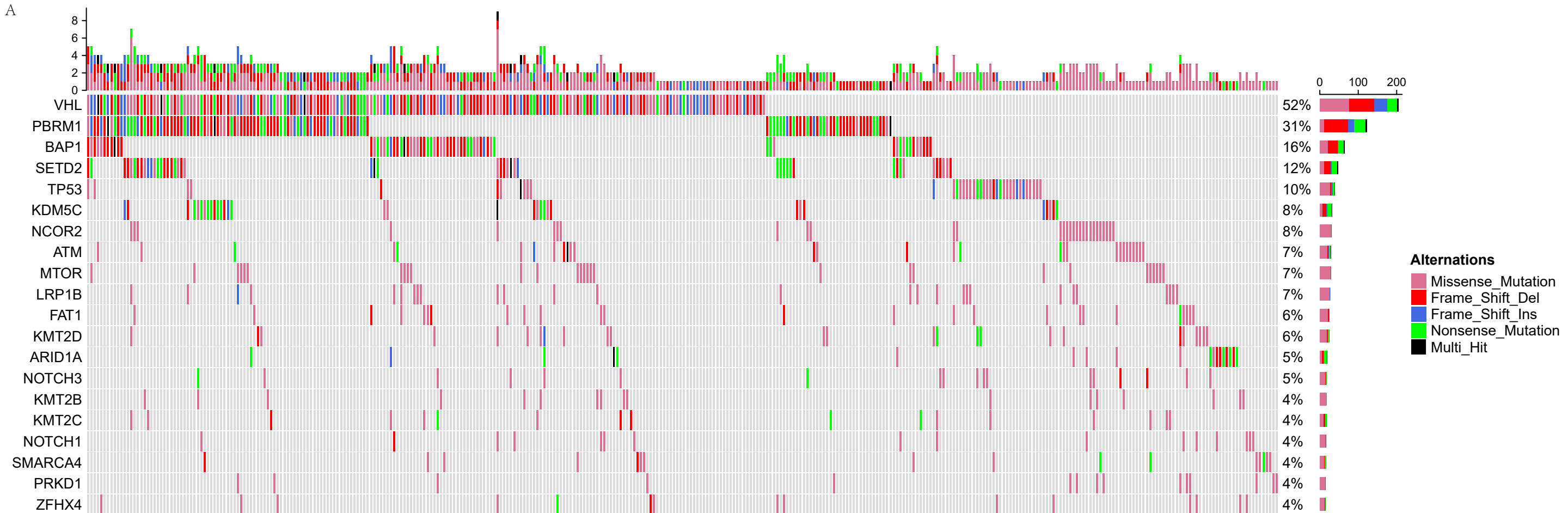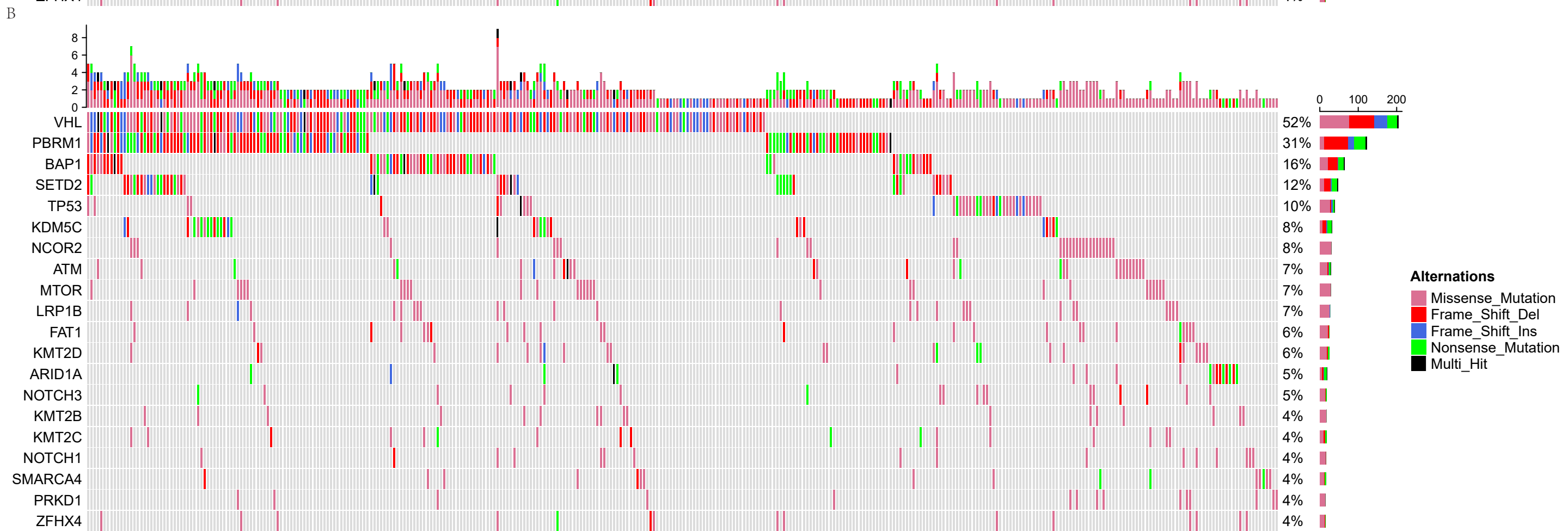

Supplement: Supplementary file 2 — Additional file 2: Figure S2. Mutational landscape between DDR-mut (A) and DDR-wt group (B). [file 40001_2024_1678_MOESM2_ESM.pdf]

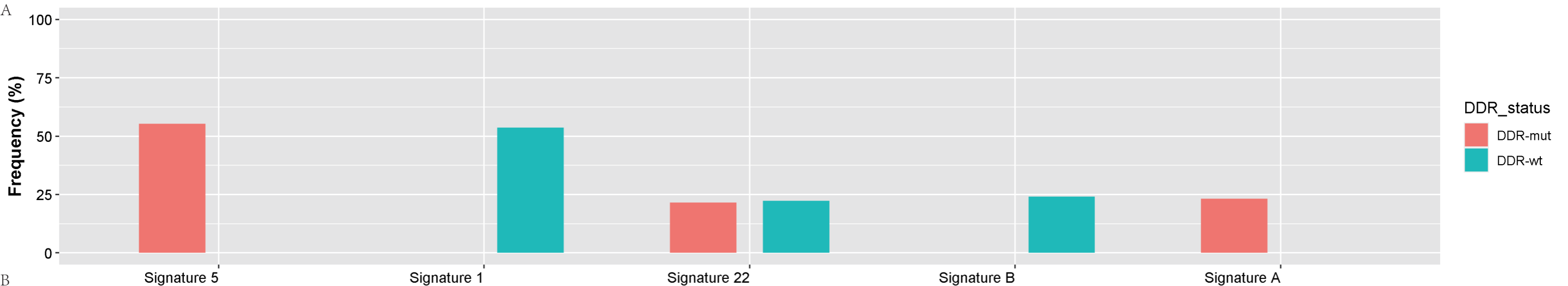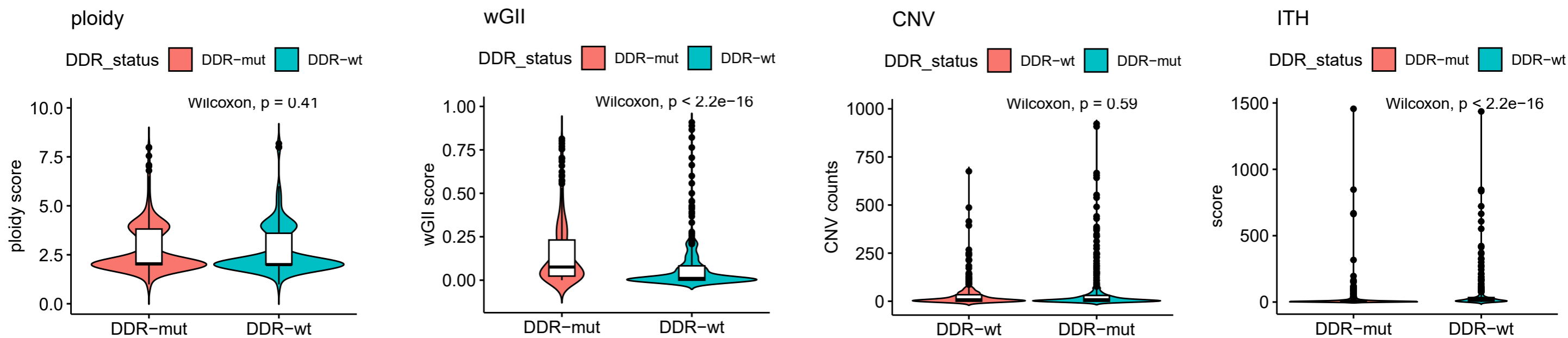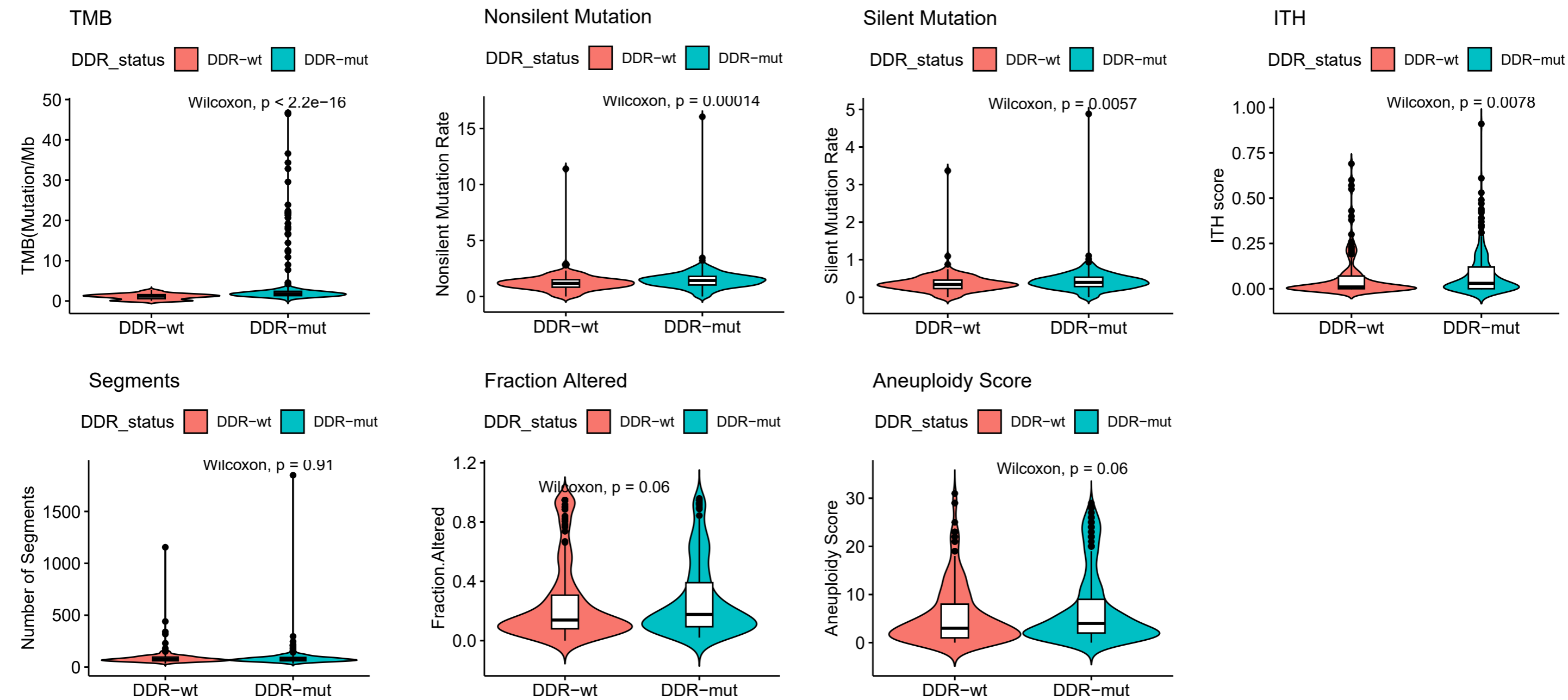

Supplement: Supplementary file 3 — Additional file 3: Figure S3. Molecular characteristics of DDR gene mutations. (A) The proportion of signatures in various DDR status. (B) The relationship between genomic instability and DDR status in the Chinses cohort. (C) The relationship between genomic instability and DDR status in the Chinses cohort. Evaluation of TMB, nonsilent mutation, and number of segments using −log(TMB), −log(nonsilent mutation), and −log(number of segments). [file 40001_2024_1678_MOESM3_ESM.pdf]

DDR\_status 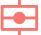 DDR-mut 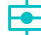 DDR-wt

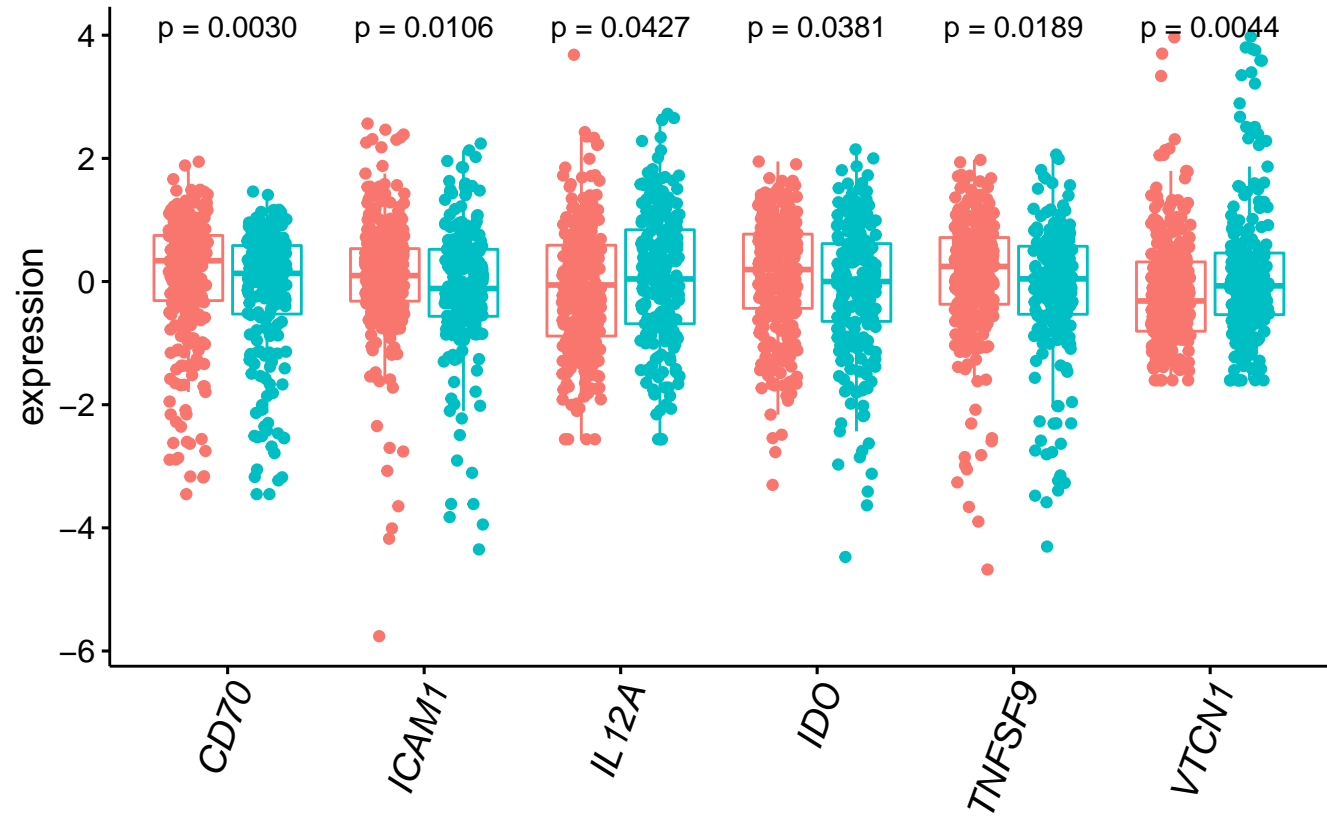

Supplement: Supplementary file 4 — Additional file 4: Figure S4. Immune-related genes were significantly differentially expressed in DDR-mut and DDR-wt. [file 40001_2024_1678_MOESM4_ESM.pdf]
