# Supplementary material for: DNA damage response alterations in clear cell renal cell carcinoma: clinical, molecular, and prognostic implications
Source: Eur J Med Res. 2024 Feb 7;29:107. doi: 10.1186/s40001-024-01678-x (PMC10848511; doi:10.1186/s40001-024-01678-x)
Supplement: Supplementary file 5 — Additional file 5: Table S1. Characteristics of Chinese kidney cancer patients. [file 40001_2024_1678_MOESM5_ESM.docx]

**Table S1 Characteristics of Chinese kidney cancer patients**

|  | Chinese cohort | | | | TCGA cohort | | | |
| --- | --- | --- | --- | --- | --- | --- | --- | --- |
| Characteristics | Overall, N = 757 | DDR-mut, N = 395 | DDR-wt, N = 362 | p-value | Overall, N = 537 | DDR-mut, N = 304 | DDR-wt, N = 233 | p-value |
| **SEX** |  |  |  | 0.213 |  |  |  | 0.636 |
| Female | 257 (33.95%) | 126 (31.90%) | 131 (36.19%) |  | 191 (35.57%) | 109 (35.86%) | 82 (35.19%) |  |
| Male | 500 (66.05%) | 269 (68.10%) | 231 (63.81%) |  | 346  (64.43%) | 195 (64.14%) | 151 (64.38%) |  |
| **AGE** | 55 | 56 | 53 | **0.005** | 61 | 61 |  | 0.214 |
| **Sample type** |  |  |  | **<0.001** |  |  |  |  |
| ctDNA | 138 (18.23%) | 26 (6.58%) | 112 (30.94%) |  | 0 | 0 | 0 |  |
| Tissue | 619 (81.77%) | 369 (93.42%) | 250 (69.06%) |  | 537 (100%) | 304(100%) | 23(100%) |  |
| **History of target drug** |  |  |  | **0.007** | / | / | / |  |
| No | 629 (83.09%) | 340 (86.08%) | 289 (79.83%) |  | / | / | / |  |
| Yes | 70 (9.25%) | 24 (6.08%) | 46 (12.71%) |  | / | / | / |  |
| Missing | 58 (7.66%) | 31 (7.85%) | 27 (7.46%) |  | / | / | / |  |
| **History of chemotherapy** |  |  |  | 0.368 | / | / | / |  |
| No | 664 (87.71%) | 347 (87.85%) | 317 (87.57%) |  | / | / | / |  |
| Yes | 10 (1.32%) | 3 (0.76%) | 7 (1.93%) |  | / | / | / |  |
| Missing | 83 (10.96%) | 45 (11.39%) | 38 (10.50%) |  | / | / | / |  |
| **History of immunotherapy** |  |  |  | 0.85 | / | / | / |  |
| No | 659 (87.05%) | 343 (86.84%) | 316 (87.29%) |  | / | / | / |  |
| Yes | 15 (1.98%) | 7 (1.77%) | 8 (2.21%) |  | / | / | / |  |
| Missing | 83 (10.96%) | 45 (11.39%) | 38 (10.50%) |  | / | / | / |  |
| **STAGE** |  |  |  |  |  |  |  | 0.474 |
| Stage I |  |  |  |  | 269 (50.09%) | 143 (47.04%) | 126 (54.08%) |  |
| Stage II |  |  |  |  | 57 (10.61%) | 35 (11.51%) | 22 (9.44%) |  |
| Stage III |  |  |  |  | 125 (23.28%) | 75 (24.67%) | 50 (21.46%) |  |
| Stage IV |  |  |  |  | 83 (15.46%) | 50 (16.45%) | 33 (14.16%) |  |
| Missing |  |  |  |  | 3 (0.56%) | 1 (0.33%) | 2 (0.86%) |  |
| **GRADE** |  |  |  |  |  |  |  | 0.672 |
| G1 |  |  |  |  | 14 (2.61%) | 7 (2.30%) | 7 (3.00%) |  |
| G2 |  |  |  |  | 230 (42.83%) | 125 (41.12%) | 105 (45.06%) |  |
| G3 |  |  |  |  | 207 (38.55%) | 119 (39.14%) | 88 (37.77%) |  |
| G4 |  |  |  |  | 78 (14.53%) | 48 (15.79%) | 30 (12.88%) |  |
| GX |  |  |  |  | 5 (0.93%) | 4 (1.32%) | 1 (0.43%) |  |
| Missing |  |  |  |  | 3 (0.56%) | 1 (0.33%) | 2 (0.86%) |  |
| 1Median (IQR) or Frequency (%) | | | | |  |  |  |  |
| 2Pearson's Chi-squared test; Wilcoxon rank sum test; Fisher's exact test | | | | |  |  |  |  |
