# Supplementary material for: DNA damage response alterations in clear cell renal cell carcinoma: clinical, molecular, and prognostic implications
Source: Eur J Med Res. 2024 Feb 7;29:107. doi: 10.1186/s40001-024-01678-x (PMC10848511; doi:10.1186/s40001-024-01678-x)
Supplement: Supplementary file 6 — Additional file 6: Table S2. The significantly different gene between the DDR-mut and DDR-wt groups in Chinese cohort. [file 40001_2024_1678_MOESM6_ESM.docx]

Table S2 The significantly different gene between the DDR-mut and DDR-wt groups in Chinese cohort

|  | DDR_mut(%) | DDR_wt(%) | FDR |
| --- | --- | --- | --- |
| VHL | 55.19 | 19.61 | 0 |
| PBRM1 | 33.67 | 0.00 | 0 |
| BAP1 | 17.22 | 0.00 | 0 |
| SETD2 | 12.15 | 1.93 | 0 |
| TP53 | 10.89 | 0.00 | 0 |
| KDM5C | 8.10 | 1.93 | 0.008 |
| ATM | 7.59 | 0.00 | 0 |
| MTOR | 7.34 | 0.00 | 0 |
| KMT2D | 6.58 | 1.38 | 0.018 |
| ARID1A | 5.06 | 0.00 | 0 |
| NOTCH1 | 4.05 | 0.28 | 0.018 |
| SMARCA4 | 4.05 | 0.00 | 0.003 |
| PTEN | 3.80 | 0.00 | 0.005 |
| NLRC5 | 3.54 | 0.28 | 0.039 |
| MSH6 | 3.29 | 0.00 | 0.017 |
| STAG2 | 3.29 | 0.00 | 0.017 |
| BRCA1 | 3.04 | 0.00 | 0.025 |
| BRCA2 | 3.04 | 0.00 | 0.025 |
| ARID1B | 2.78 | 0.00 | 0.039 |
| ATRX | 2.78 | 0.00 | 0.039 |
| MDC1 | 2.78 | 0.00 | 0.039 |
| MLH1 | 2.78 | 0.00 | 0.039 |
