# Supplementary material for: DNA damage response alterations in clear cell renal cell carcinoma: clinical, molecular, and prognostic implications
Source: Eur J Med Res. 2024 Feb 7;29:107. doi: 10.1186/s40001-024-01678-x (PMC10848511; doi:10.1186/s40001-024-01678-x)
Supplement: Supplementary file 7 — Additional file 7: Table S3. The significantly different gene between the DDR-mut and DDR-wt groups in TCGA cohort. [file 40001_2024_1678_MOESM7_ESM.docx]

Table S3 The significantly different gene between the DDR-mut and DDR-wt groups in TCGA cohort

|  | DDR_mut (%) | DDR_wt (%) | FDR |
| --- | --- | --- | --- |
| VHL | 51.64 | 29.18 | 0.000 |
| PBRM1 | 45.07 | 0.00 | 0.000 |
| TTN | 19.41 | 4.72 | 0.000 |
| MUC16 | 16.78 | 3.00 | 0.000 |
| BAP1 | 12.17 | 0.00 | 0.000 |
| MTOR | 10.53 | 0.00 | 0.000 |
| XIRP2 | 8.22 | 1.29 | 0.046 |
| ARID1A | 8.22 | 0.00 | 0.001 |
| TSHZ3 | 8.22 | 0.00 | 0.001 |
| ARAP3 | 7.89 | 0.43 | 0.007 |
| SSH2 | 7.57 | 0.43 | 0.011 |
| NAV3 | 7.24 | 0.43 | 0.016 |
| CD4 | 7.24 | 0.00 | 0.004 |
| GRIN2B | 6.91 | 0.43 | 0.021 |
| MAP1B | 6.91 | 0.43 | 0.021 |
| ABCA6 | 6.91 | 0.00 | 0.004 |
| DNMT1 | 6.91 | 0.00 | 0.004 |
| FAM151A | 6.91 | 0.00 | 0.004 |
| HEBP1 | 6.91 | 0.00 | 0.004 |
| SSX3 | 6.91 | 0.00 | 0.004 |
| LRP1 | 6.58 | 0.43 | 0.033 |
| FANCE | 6.58 | 0.00 | 0.006 |
| MYO3B | 6.58 | 0.00 | 0.006 |
| PIAS3 | 6.58 | 0.00 | 0.006 |
| SEC24C | 6.58 | 0.00 | 0.006 |
| CACNA1S | 6.25 | 0.43 | 0.046 |
| PAPPA2 | 6.25 | 0.43 | 0.046 |
| PHACTR1 | 6.25 | 0.43 | 0.046 |
| TSKS | 6.25 | 0.43 | 0.046 |
| CR2 | 6.25 | 0.00 | 0.009 |
| PCDH12 | 6.25 | 0.00 | 0.009 |
| PTEN | 6.25 | 0.00 | 0.009 |
| ARFGEF1 | 5.92 | 0.00 | 0.013 |
| ATP2B4 | 5.92 | 0.00 | 0.013 |
| DSCAM | 5.92 | 0.00 | 0.013 |
| EP300 | 5.92 | 0.00 | 0.013 |
| KIAA0408 | 5.92 | 0.00 | 0.013 |
| RANBP2 | 5.92 | 0.00 | 0.013 |
| UNC5C | 5.92 | 0.00 | 0.013 |
| BAG6 | 5.59 | 0.00 | 0.018 |
| EIF3B | 5.59 | 0.00 | 0.018 |
| GLI1 | 5.59 | 0.00 | 0.018 |
| ITGA10 | 5.59 | 0.00 | 0.018 |
| ITGAV | 5.59 | 0.00 | 0.018 |
| NEUROD1 | 5.59 | 0.00 | 0.018 |
| NOMO1 | 5.59 | 0.00 | 0.018 |
| NRSN2 | 5.59 | 0.00 | 0.018 |
| OR10G8 | 5.59 | 0.00 | 0.018 |
| SLPI | 5.59 | 0.00 | 0.018 |
| ALOX12 | 5.26 | 0.00 | 0.027 |
| C1S | 5.26 | 0.00 | 0.027 |
| CA10 | 5.26 | 0.00 | 0.027 |
| CARD8 | 5.26 | 0.00 | 0.027 |
| EPHX4 | 5.26 | 0.00 | 0.027 |
| FKBP9 | 5.26 | 0.00 | 0.027 |
| KLK1 | 5.26 | 0.00 | 0.027 |
| SMG7 | 5.26 | 0.00 | 0.027 |
| BPIFC | 4.93 | 0.00 | 0.042 |
| C6 | 4.93 | 0.00 | 0.042 |
| CCDC136 | 4.93 | 0.00 | 0.042 |
| CCDC91 | 4.93 | 0.00 | 0.042 |
| HERC2 | 4.93 | 0.00 | 0.042 |
| MRPL24 | 4.93 | 0.00 | 0.042 |
| NAV1 | 4.93 | 0.00 | 0.042 |
| PIGO | 4.93 | 0.00 | 0.042 |
| SLC1A3 | 4.93 | 0.00 | 0.042 |
| SREBF2 | 4.93 | 0.00 | 0.042 |
| TBX6 | 4.93 | 0.00 | 0.042 |
| TP53 | 4.93 | 0.00 | 0.042 |
